# Strong field gradients enable NMR-based diffusion measurements for K^+^, Mg^2+^, Cl^−^, and ${\text{SO}}_{4}^{2-}$ ions in biomolecular solutions

**DOI:** 10.1016/j.jmr.2025.107890

**Published:** 2025-04-30

**Authors:** Tianzhi Wang, Daniel Arcos, F. David Doty, B. Montgomery Pettitt, Junji Iwahara

**Affiliations:** a Department of Biochemistry & Molecular Biology, Sealy Center for Structural Biology & Molecular Biophysics, University of Texas Medical Branch, Galveston, TX 77555-1068, USA; b Doty Scientific, Inc., Columbia, SC 29229, USA

**Keywords:** Dynamics, Electrostatics, Ions, Nuclear magnetic resonance, Pulsed field gradients, Quadrupolar nuclei

## Abstract

NMR-based diffusion measurements of potassium (K^+^), magnesium (Mg^2+^), chloride (Cl^−^), and sulfate (${\text{SO}}_{4}^{2-}$) ions have been challenging even though these ions are biologically important. For these ions, the gyromagnetic ratios of the NMR-active nuclei, ^39^K, ^25^Mg, ^35^Cl, and ^33^S, are less than 1/10 of the ^1^H gyromagnetic ratio, causing a low sensitivity in NMR detection and a low efficiency in NMR dephasing needed for diffusion measurements. These nuclei also undergo rapid longitudinal and transverse NMR relaxation via the quadrupolar mechanism, severely limiting the effectiveness of NMR-based diffusion measurements. Interactions with biomolecules promote the NMR relaxation of these ions, hindering measurements of the ion diffusion. We demonstrate that, despite these challenges, diffusion of K^+^, Mg^2+^, Cl^−^, and ${\text{SO}}_{4}^{2-}$ ions in biomolecular solutions can be measured accurately and precisely through use of appropriately designed high-field NMR probe hardware that can generate strong field gradients >1000 G/cm. The NMR-based diffusion coefficients measured at 17.6 T for these ions in the absence of biomolecules agreed well with conductivity-based values in the literature. This consistency supports that ion diffusion along the magnetic field is unaffected by the Lorentz force acting on the ions, as previously predicted. Our data on ion diffusion in solutions of proteins and DNA illuminate the effect of electrostatic interactions on the apparent diffusion coefficients of ions. Thus, high-field NMR probe hardware that can generate strong field gradients opens a new avenue to characterize the dynamic behavior of various ions around biomolecules and their effect on biomolecular electrostatics.

## 1. Introduction

Nuclear magnetic resonance (NMR) spectroscopy is powerful for investigating molecular diffusion in liquid [1–3]. Stimulated-echo (STE) methods using pulsed-field-gradients (PFGs) are particularly effective for obtaining quantitative information on translational diffusion, allowing for accurate determination of diffusion coefficients for various solutes, including small molecules and macromolecules [1–3]. Unlike other methods, NMR-based diffusion methods can provide information about the diffusion of particular molecules in multi-component mixtures without requiring labeling or chemical modification. Since the diffusional properties depend on not only hydrodynamic radii but also molecular interactions and aggregation, NMR-based diffusion measurements are useful for characterizing various small- and macromolecules [4–6].

Ion diffusion can also be characterized through NMR-based measurements [7–9]. Unlike conductivity-based measurement of ion diffusion, NMR-based measurement can be applied to investigate the diffusional behavior of ions in more complex solutions, including those containing biological macromolecules. In previous studies, diffusion of sodium (Na^+^), ammonium (NH4^+^), and acetate (CH_3_COO^−^) ions acting as counterions of proteins and DNA were studied using ^23^Na, ^15^N, and ^13^C diffusion NMR methods, respectively [10–12]. Through ^7^Li and ^133^Cs diffusion NMR, the diffusional properties of Li^+^ and Cs^+^ ions around DNA were also compared with those of Na^+^ ions [13].

NMR-based diffusion measurements are more challenging for other ions important for biological systems such as potassium (K^+^), magnesium (Mg^2+^), chloride (Cl^−^), and sulfate (${\text{SO}}_{4}^{2-}$) ions. The NMR-active nuclei for these ions, ^39^K, ^25^Mg, ^35^Cl, and ^33^S, are quadrupolar nuclei with a small gyromagnetic ratio γ. Their γ ratios are less than 1/10 of the ^1^H γ ratio, as shown in Table 1. The small γ values of these nuclei impose several practical problems on NMR-based diffusion measurements. A problem is that the ^25^Mg, ^33^S, ^35^Cl, and ^39^K NMR frequencies are too low to be covered by typical broadband probe hardware. More fundamental problems of these low-γ nuclei are the low sensitivity in NMR detection and the low efficiency in NMR dephasing for diffusion measurements. Furthermore, ^39^K, ^25^Mg, ^35^Cl, and ^33^S nuclei are quadrupolar nuclei that exhibit rapid NMR relaxation, and their relaxation is remarkably faster in biomolecular solutions where the ions can interact with biomolecules, as previously shown [14–18] and further demonstrated in our current work. Such rapid quadrupolar relaxation for both longitudinal and transverse magnetizations severely limits the applicability of the stimulated-echo method, posing another challenge in diffusion NMR measurements [1].

In this paper, we demonstrate that despite the aforementioned challenges, diffusion of K^+^, Mg^2+^, Cl^−^, and ${\text{SO}}_{4}^{2-}$ ions in biomolecular solutions can be accurately and precisely measured through the use of appropriately designed high-field NMR probe hardware that can generate PFGs >1000 G/cm, which are substantially stronger than the maximum PFGs of typical NMR probes (~55 G/cm). The probe hardware used in this study is equipped with a PFG coil that can generate up to 2700 G/cm (with a 60-A gradient amplifier) and a multinuclear RF coil circuit allowing insertion of appropriate tuning and matching capacitors for ^39^K, ^25^Mg, ^35^Cl, or ^33^S nuclei. Through comparison with literature data on conductivity-based diffusion coefficients of ions in the absence of biomolecules, we have also confirmed that ion diffusion measurement by NMR is unaffected by the Lorentz force acting on ions under the high magnetic field. Our data show that electrostatic interactions with biomolecules make the ion diffusion slower, especially for divalent ions. Thus, the ion NMR methods utilizing strong PFGs are useful for investigating ion diffusion and electrostatics in biomolecular systems.

## 2. Experimental

### 2.1. Materials

Chemical reagents were purchased from Sigma-Aldrich (St. Louis, MO, USA). The biomolecular samples of a 12-bp DNA duplex (with the nucleotide sequence of AGCGTGGGCGTA), the Egr-1 DNA-binding domain (DBD; zinc fingers 1–3) and the Antp homeodomain were prepared as previously described [20,21]. For NMR samples, 5-mm tubes (cat.# S5–600-7) and 2-mm coaxial stem inserts (cat.# NI5CCI-B) were purchased from Norell (Morganton, NC, USA).

NMR spectrometer and software.

All NMR experiments were performed at 25 °C using a Bruker Avance III 750-MHz spectrometer with an Oxford narrow-bore superconducting magnet operated at the magnetic field of 17.6 T. TopSpin^™^ version 3.6 was used to operate the NMR spectrometer. The sample temperature was calibrated using a standard method with methanol [22]. NMR data were processed with TopSpin and NMR-Pipe [23]. An exponential window function with a broadening factor of 3 Hz was used prior to the Fourier transform for each free induction decay (FID). Nonlinear least-squares fitting calculations were performed using in-house MATLAB (Math Works, Inc.) scripts using the ‘nlinfit’ function.

### 2.2. Diffusion NMR probe hardware

For ^25^Mg, ^33^S, ^35^Cl, and ^39^K diffusion NMR experiments, a Doty 750-MHz *z*-gradient diffusion probe (‘DSI-1699’) was used together with a Bruker GREAT60 amplifier that can produce currents up to 60 A. This 5-mm probe has two RF coils together with an actively shielded *z*-axis PFG coil. The inner multinuclear RF coil is essentially the classic Zens compensated-foil coil but with a center tap to ground (through a small capacitor) to prevent problematic modes near ^1^H that otherwise could likely arise when tuning a high inductance coil (here ~150 nH) to a low-gamma nuclide. Observing nuclides such as ^25^Mg, ^33^S, ^35^Cl, or ^39^K involved the insertion of appropriate tuning and matching capacitors through “chip-on-a-stick” wands, which were designed for the nuclide of interest. One of the uncommon features of the multinuclear rf circuit is the use of 2–16 pF high-voltage variable capacitors so there is plenty of adjustment range when tuned to low frequencies. The outer rf coil is a compensated-foil saddle resonator double-tuned for ^1^H and ^2^H. Externally accessible knobs for the tuning and matching variable capacitors were used for fine adjustment for each sample. The PFG coil (the inner and outer diameters being 16 mm and 38 mm, respectively), consisted of multiple layers of windings on alumina ceramic coilformers to handle the high currents with minimal vibrations. It has a resistance of 1.7 Ω and an inductance of 158 μH. The PFG coil was cooled with water circulated at 25 °C to avoid overheating. The DSI-1699 probe is designed to achieve 4 % root-mean-square (RMS) gradient uniformity over a cylindrical space with a 12-mm height (*H*) and a 7-mm diameter (*ϕ*); and 1 % RMS gradient uniformity over a 9 mm (*H*) × 5 mm (*ϕ*) cylinder. The multinuclear RF coil length is 12 mm to match the high homogeneity region of the PFG coil. The probe generates a magnetic field gradient magnitude of 0.45 T/(A•m), allowing for a gradient strength of 2700 G/cm at 60 A. PFG coil temperature was monitored with an internal thermocouple connected to the master unit that controls the GREAT60 gradient amplifier and protects the probe by halting the experiment whenever the PFG coil overheating is detected.

### 2.3. Diffusion measurements

All ions diffusion measurements were conducted through the bipolar-pair longitudinal-eddy-current-delay (BPP-LED) stimulated-echo (STE) experiments [24,25] using the Bruker pulse sequence ‘stebpgp1s’ together with PFGs with a smoothed square shape (‘SMSQ10’). For the BPP-LED STE data, each diffusion coefficient *D* was determined from the signal intensities (*I*) as a function of the magnetic field gradient strength (*g*) through nonlinear least-squares fitting using the following function [26]:

(1) $$I\left(g\right)=I_{0}\exp\left[-D\gamma^{2}g^{2}\sigma^{2}\delta^{2}\left(\text{Δ}+\frac{\left(2\kappa -2\lambda -1\right)\delta }{4}-\frac{\tau }{2}\right)\right]$$

In Eq. 1, *I*_0_ is an intrinsic intensity with no attenuation by diffusion; Δ is the total duration between the first 90° pulses of the two bipolar pairs; *τ* is the delay between each PFG and the following RF pulse; and *δ* is the length of each PFG in the bipolar schemes. The parameters *σ*, *κ*, and *λ* in Eq. 1 are specific to the PFG shape. For the smoothed rectangle shape ‘SMSQ10’, the values, *σ* = 0.9, *λ* = 0.5, and *κ* = 0.3495, were taken from Table 1 of Sinnaeve [26]. The PFG strengths listed in ‘difflist’ created by TopSpin software corresponds to *gσ* in Eq. 1. When the up/down ramps of the PFG shape are as short as 0.1*δ* (which is the case for SMSQ10), Eq. 1 remains highly accurate for a wide range of Δ/*δ* [27], including the conditions in the current study.

Gradient strengths for the aforementioned NMR probe were calibrated through bipolar stimulated-echo experiments on the self-diffusion coefficient of *N*,*N*-dimethylformamide (DMF), which is 1.63 × 10^9^ m^2^ s^−1^ at 25 °C [28]. For this calibration, 380 μL of DMF was placed into a 5-mm NMR tube together with a 2-mm coaxial stem insert containing 100 μL of D_2_O for NMR lock. The annular geometry of the coaxial tube suppress convection that may adversely affect diffusion measurements [29]. Experimental conditions for measuring diffusion of ^35^Cl^−^, ^39^K^+^, ^25^Mg^2+^, and SO42−33 ions are indicated below. For all diffusion experiments, the recycle delay was typically set to 0.7 s. Although it was far longer than 5*T*_1_ for some cases of these quadrupolar nuclei (see below), this relatively long recycle delay was set due to the duty cycle limit for the PFG coil that generates strong gradients.

### 2.4. NMR relaxation measurements

Apparent *T*_1_ and *T*_2_ relaxation times of ^25^Mg, ^33^S, ^35^Cl, and ^39^K nuclei were measured through the inversion recovery experiments and line-shape fitting, respectively. To determine a longitudinal relaxation time *T*_1_, the following function was used for the nonlinear least-squares fitting to inversion recovery data:

(2) $$I(t)=\left(I_{o}-I_{B}\right)exp\left(-t/T_{1}\right)+I_{B},$$

where *t* represents a delay for longitudinal relaxation; *I_o_* is the initial intensity; and *I_B_* is the signal intensity for the magnetization at Boltzmann equilibrium. Since rapid quadrupolar relaxation governs NMR line-shape for quadrupolar nuclei, the line-shape fitting can be used to assess apparent transverse relaxation times for the current case with quadrupolar ^25^Mg, ^33^S, ^35^Cl, and ^39^K nuclei. To determine an apparent transverse relaxation time *T*_2_*, the following Lorentzian function was used for the nonlinear least-squares fitting to a 1D NMR spectrum:

(3) $$A(v)=\frac{a_{o}R_{2,app}}{4\pi^{2}{\left(v-v_{a}\right)}^{2}+R_{2,app}^{2}},$$

where *ν* is a chemical shift in Hz units; *ν_a_* is the peak position; *a_0_* is an intrinsic amplitude; and *R*_2*,app*_ is the apparent transverse relaxation rates. Since *R*_2_*,_app_* includes the effect of an exponential window function with a line-broadening factor (*L_B_* in Hz units), the apparent transverse relaxation time *T*_2_* was calculated as 1/(*R*_2*,app*_ – π*L_B_*).

### 2.5. ^35^Cl NMR experiments for chloride ions

To investigate interactions between Cl^−^ ions and the Egr-1 DBD, we conducted ^35^Cl NMR experiments for two solutions: 1) 1 mM Egr-1 DBD solution in a buffer of 20 mM succinate-d_4_•KOH (pH 5.8), 20 mM KCl, and 0.1 mM ZnCl_2_; and 2) the buffer only. For each, the sample volume was 380 μL, which was placed in a 5-mm NMR tube together with a 2-mm coaxial stem insert containing 100 μL of D_2_O for NMR lock. The RF strength for ^35^Cl pulses was 9.84 kHz. In the ^35^Cl BPP-LED STE experiments, conditions with Δ = 8 ms, *δ* = 1 ms, *τ* = 1.2 ms, and 11 different PFG strengths up to 853 G/cm were used to measure diffusion. 12,288 scans were accumulated per FID.

### 2.6. ^39^K NMR experiments for potassium ions

The ^39^K NMR experiments were conducted for two 5-mm NMR tube samples. One of them contained a 520 μL solution of 1 mM 12-bp DNA duplex in a buffer of 20 mM Tris•HCl (pH 7.5), 100 mM KCl, and 5 % D_2_O. The other sample was a control sample with no DNA in the same buffer. The RF strength for ^39^K pulses was 7.81 kHz. The diffusion coefficients of ^39^K^+^ ions were measured through the BPP-LED STE experiments with Δ = 9.6 ms, *δ* = 1.1 ms, and *τ* = 1.5 ms. Eleven different PFG strengths up to 1454 G/cm were used. 4400 scans were accumulated per FID.

### 2.7. ^25^Mg NMR experiments for magnesium ions

The NMR experiments for Mg^2+^ ions were conducted for natural-abundance ^25^Mg nuclei in the following two samples. One of them contained a 520 μL solution of 1 mM 12-bp DNA duplex in a buffer of 20 mM Tris•HCl (pH 7.5), 100 mM MgCl_2_, and 5 % D_2_O. The other sample was a control sample with no DNA in the same buffer. These samples were sealed in 5-mm NMR tubes. The RF strength for ^25^Mg pulses was 8.28 kHz. To measure diffusion, the ^25^Mg BPP-LED STE experiments were conducted with Δ = 10 ms, *δ* = 1.5 ms, *τ* = 1.5 ms, and 11 different PFG strengths up to 1279 G/cm. 12,228 scans were accumulated per FID.

### 2.8. ^33^S NMR experiments for sulfate ions

Because the natural abundance of ^33^S is only 0.76 %, NMR experiments for ${\text{SO}}_{4}^{2-}$ ions were conducted using sodium sulfate-^33^S (98 %; Sigma-Aldrich, cat.# 719,374). Two 5-mm tube samples were used: 1) a 520 μL solution of 0.4 mM Antp HD in a buffer of 20 mM Tris•HCl (pH 7.5), 10 mM sodium sulfate-^33^S, and 5 % D2O; and 2) a 520 μL sample of the same buffer. The RF strength for ^33^S pulses was 9.26 kHz. In the ^33^S BPP-LED STE experiments Δ = 10 ms, *δ* = 1.5 ms, *τ* = 1.2 ms, and 11 different PFG strengths up to 853 G/cm were used to measure diffusion. 4096 scans were accumulated for each FID.

## 3. Results

We conducted NMR experiments for two cations (^39^K^+^ and ^25^Mg^2+^) and two anions (^35^Cl^−^, and SO42−33) using several biomolecular solutions. To assess the impact of interactions with the biomolecules, the NMR experiments for each type of these ions were performed in the presence and absence of the biomolecules with a large net charge opposite to the observed ions.

### 3.1. Ion NMR relaxation promoted by interactions with biomolecules

**Fig. 1.**
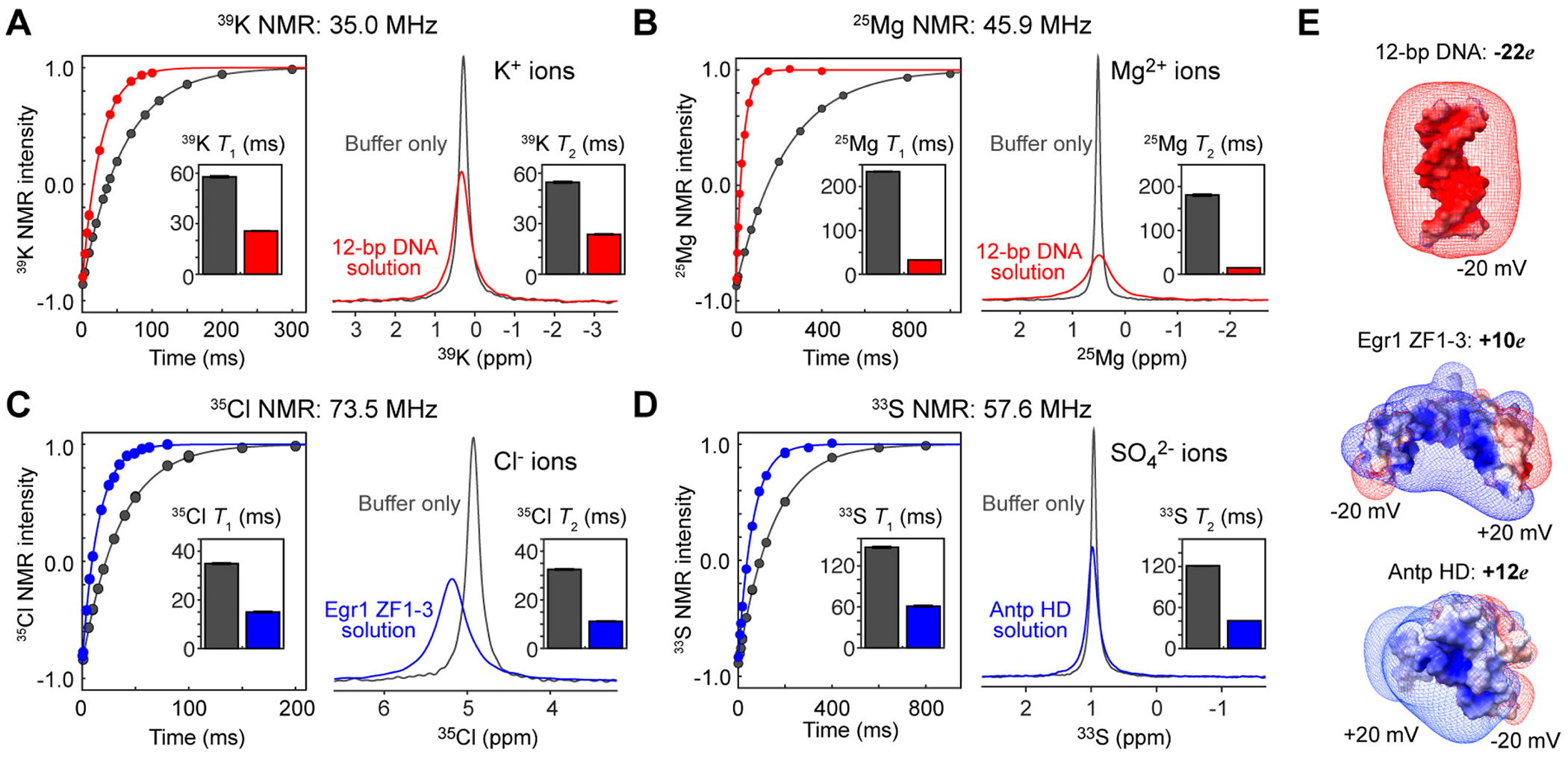
Interactions with biomolecules promote NMR relaxation of K^+^, Mg^2+^, Cl^−^, and ${\text{SO}}_{4}^{2-}$ ions. The NMR data were obtained at the magnetic field of 17.6 T, where the ^1^H frequency is 750 MHz. The inversion recovery data and the overlaid 1D NMR spectra (for a 250-Hz range containing the signals) are shown. The data for the buffer only are shown in gray. (**A**) ^39^K NMR relaxation data for K^+^ ions (100 mM) in the presence and absence of 1 mM 12-bp DNA. (**B**) ^25^Mg NMR relaxation data for Mg^2+^ ions (100 mM) in the presence and absence of 1 mM 12-bp DNA. (**C**) ^35^Cl NMR relaxation data for Cl^−^ ions (20 mM) in the presence and absence of 1 mM Egr-1 DBD. (**D**) ^33^S NMR relaxation data for ${\text{SO}}_{4}^{2-}$ ions (10 mM; ^33^S-labeled) in the presence and absence of 0.4 mM Antp homeodomain. (**E**) Isopotential maps (at ±20 mV) and overall charges of the biomolecules used in this study. The electrostatic potentials were computed at pH 7.5 and the ionic strength of 100 mM using APBS [39]. The structures were drawn with ChimeraX [40].

For ^39^K, ^25^Mg, ^35^Cl, and ^33^S nuclei, we measured apparent longitudinal (*T*_1_) and transverse (*T*_2_) relaxation times through inversion recovery experiments and the line-shape fitting, respectively (Fig. 1A–D). It should be noted that for quadrupolar nuclei, the impact of magnetic field inhomogeneity on 1/*T*_2_* is negligible compared to rapid quadrupolar relaxation. Both *T*_1_ and *T*_2_ relaxation times of the ions were considerably shorter when biomolecules with a large opposite charge were present in the same solutions. In biomolecular solutions, the ion *T*_1_ and *T*_2_ relaxation times were shorter than 60 ms for all cases. For ^39^K^+^ ions in the DNA solution and ^35^Cl^−^ ions in the protein solution, the *T*_1_ relaxation times were even shorter than 30 ms. The changes due to the biomolecules were the most drastic for ^25^Mg^2+^ ions: from 235 ms to 32 ms for *T*_1_ and from 181 to 14 ms for *T*_2_. Increases in quadrupolar NMR relaxation rates 1/*T*_1_ or 1/*T*_2_ through electrostatic interactions with biomolecules have been reported for ^39^K^+^, ^25^Mg^2+^, ^35^Cl^−^, and SO42−33 ions [14–18] as well as ^7^Li^+^, ^23^Na^+^, ^87^Rb^+^, ^133^Cs^+^ ions [11,30–38], since James and Noggle reported for ^23^Na^+^ ions interacting with RNA in 1969 [30].

The decreases in the *T*_1_ and *T*_2_ relaxation times for these ions upon their interactions with biomolecules can stem from several factors: an increase in the quadrupolar coupling due to an altered electric-field-gradient (EFG) tensor; an increase in the correlation time for the EFG tensor; and chemical exchange. The EFG tensor can be affected by the electric field of the biomolecule, whereas the EFG correlation times are impacted by transient direct association with biomolecules. Theoretical details about these effects for spin-3/2 nuclei of ions interacting with macromolecules were described by Halle and coworkers [34,37].

Rapid *T*_1_ relaxation poses a practical challenge in diffusion measurements for these quadrupolar nuclei. Although *T*_2_ relaxation is also rapid for them, it may not necessarily be problematic for measuring diffusion. In fact, typical ^1^H *T*_2_ relaxation times of small to mid-size proteins (< 40 kDa) are comparable to the *T*_2_ relaxation times observed here for the ^39^K^+^, ^25^Mg^2+^, ^35^Cl^−^, and SO42−33 ions in the biomolecular solutions, but it is straightforward to measure the diffusion of such proteins [41–43]. The large *γ* ratio and slow *T*_1_ relaxation of ^1^H magnetization allows for sufficient dephasing through relatively small PFGs and a long duration Δ. By contrast, the small *γ* ratios of ^39^K, ^25^Mg, ^35^Cl, and ^33^S nuclei reduce the efficiency in dephasing (see Eq. 1), while a long duration Δ cannot compensate for this problem because the NMR signal would completely decay through rapid *T*_1_ relaxation. Therefore, it is essential to use strong field gradients for diffusion measurements on ^39^K^+^, ^25^Mg^2+^, ^35^Cl^−^, and SO42−33 ions in biomolecular solutions.

### 3.2. Diffusion of ^39^K^+^, ^25^Mg^2+^, ^35^Cl^−^, and SO42−33 ions

**Fig. 2.**
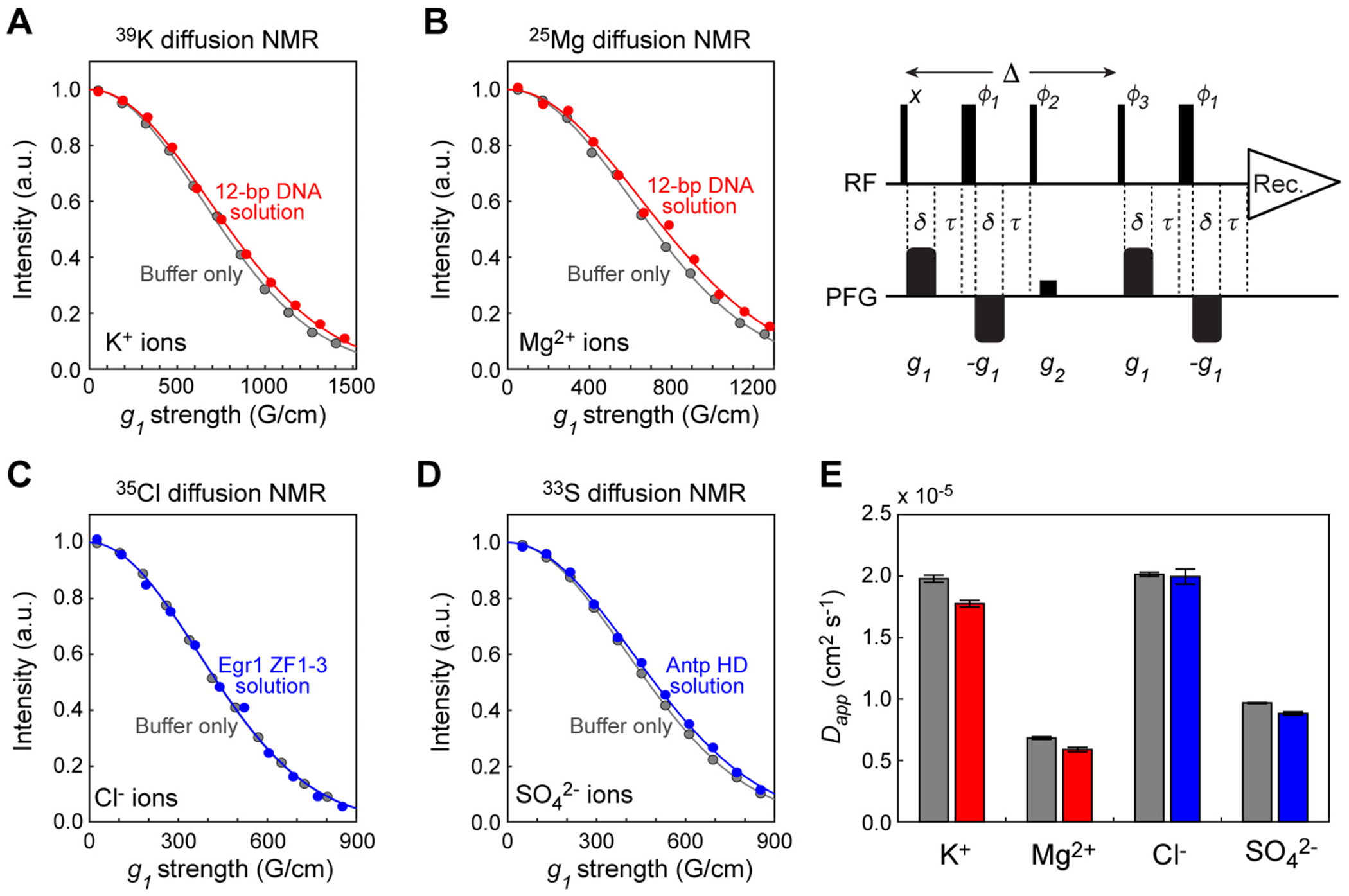
Strong PFGs enable diffusion measurements of K^+^, Mg^2+^, Cl^−^, and ${\text{SO}}_{4}^{2-}$ ions in biomolecular solutions despite rapid NMR relaxation and small gyromagnetic ratios (*γ*) of ^33^K, ^25^Mg, ^35^Cl, and ^33^S nuclei. The samples are the same as those used for Fig. 1. (**A**) ^39^K NMR-based diffusion data for K^+^ ions in the presence and absence of 1 mM 12-bp DNA. Δ = 9.6 ms, *δ* = 1.1 ms, and *τ* = 1.5 ms were used. (**B**) ^25^Mg NMR-based diffusion data for Mg^2+^ ions (100 mM) in the presence and absence of 1 mM 12-bp DNA. Δ = 10 ms, *δ* = 1.5 ms, and *τ* = 1.5 ms were used. (**C**) ^35^Cl NMR-based diffusion data for Cl^−^ ions (20 mM) in the presence and absence of 1 mM Egr-1 DBD. Δ = 8 ms, *δ* = 1 ms, and *τ* = 1.2 ms were used. (**D**) ^33^S NMR-based diffusion data for ${\text{SO}}_{4}^{2-}$ ions (10 mM; ^33^S-labeled) in the presence and absence of 0.4 mM Antp homeodomain. Δ = 10 ms, *δ* = 1.5 ms, and *τ* = 1.2 ms were used. (**E**) Diffusion coefficients measured using the data shown in Panels A-D. The data shown in gray are for the buffers only; those in red are for the 12-bp DNA solutions; and those in blue are for the protein solutions. In Panels A-D, signal intensities are shown as a function of the net gradient strengths for each SMSQ10-shape PFG in the bipolar schemes. The solid lines represent the best-fit curves obtained through nonlinear-least squared fitting using Eq. 1. The BPP-LED STE pulse sequence is also shown to indicate the definitions of the parameters Δ, *δ*, and *τ*. (For interpretation of the references to colour in this figure legend, the reader is referred to the web version of this article.)

We used the BPP-LED STE pulse sequence [24,25] (Fig. 2) to measure the diffusion of ^39^K^+^, ^25^Mg^2+^, ^35^Cl^−^, and SO42−33 ions. The bipolar pairs of PFGs can reduce the adverse effects of eddy currents [24,25,43]. This feature is important for our current experiments with strong PFG strengths up to ~1500 G/cm. To avoid a major loss due to rapid relaxation, we used a duration Δ of 8–10 ms in the ^39^K/^25^Mg/^35^Cl/^33^S diffusion NMR experiments. The strong PFG strengths allowed us to achieve significant diffusion-induced attenuation of signals, as shown in Fig. 2A–D.

Using Eq. 1, we determined the diffusion coefficients of ^39^K^+^, ^25^Mg^2+^, ^35^Cl^−^, and SO42−33 ions from the observed signal intensities as a function of the PFG strength. In each case, the measurement was replicated three or more times. As shown in Fig. 2E, the apparent diffusion coefficients *D_app_* were precisely determined for these ions in the biomolecular solutions as well as in the control samples with the buffers only. The *D_app_* coefficients for the ions in the biomolecular solutions were smaller than those for the ions in the control samples with the buffers only. The decreases in the *D_app_* coefficients in the presence of the biomolecules reflect the electrostatic interactions between the ions and the biomolecules. The decrease was more significant for the divalent ions (^25^Mg^2+^ and SO42−33) than the monovalent ions (^39^K^+^ and ^35^Cl^−^). As explained in previous studies on ions in biomolecular solutions [10–13], the apparent diffusion coefficient *D_app_* is given by (1 – *p_b_*)*D_f_* + *p_b_D_b_*, where *D_f_* is the intrinsic diffusion coefficient for the free ions; *D_b_* is a diffusion coefficient for ions within the ‘ion atmosphere’ in which ions electrostatically interact with a biomolecule; and *p_b_* is the fraction of the ions within the ion atmosphere. Although the determination of *p_b_* and *D_b_* through more extensive experiments is possible, such experiments are beyond the scope of this paper, and more detailed characterizations of ion-protein and ion-DNA interactions will be reported elsewhere. The significance of the data shown in Fig. 2 is that these results clearly demonstrate that strong field gradients enable measurements of diffusion of K^+^, Mg^2+^, Cl^−^, and ${\text{SO}}_{4}^{2-}$ ions in biomolecular solutions despite several challenges mentioned in the Introduction.

### 3.3. Assessment of Lorentz force’s influence on diffusion

**Fig. 3.**
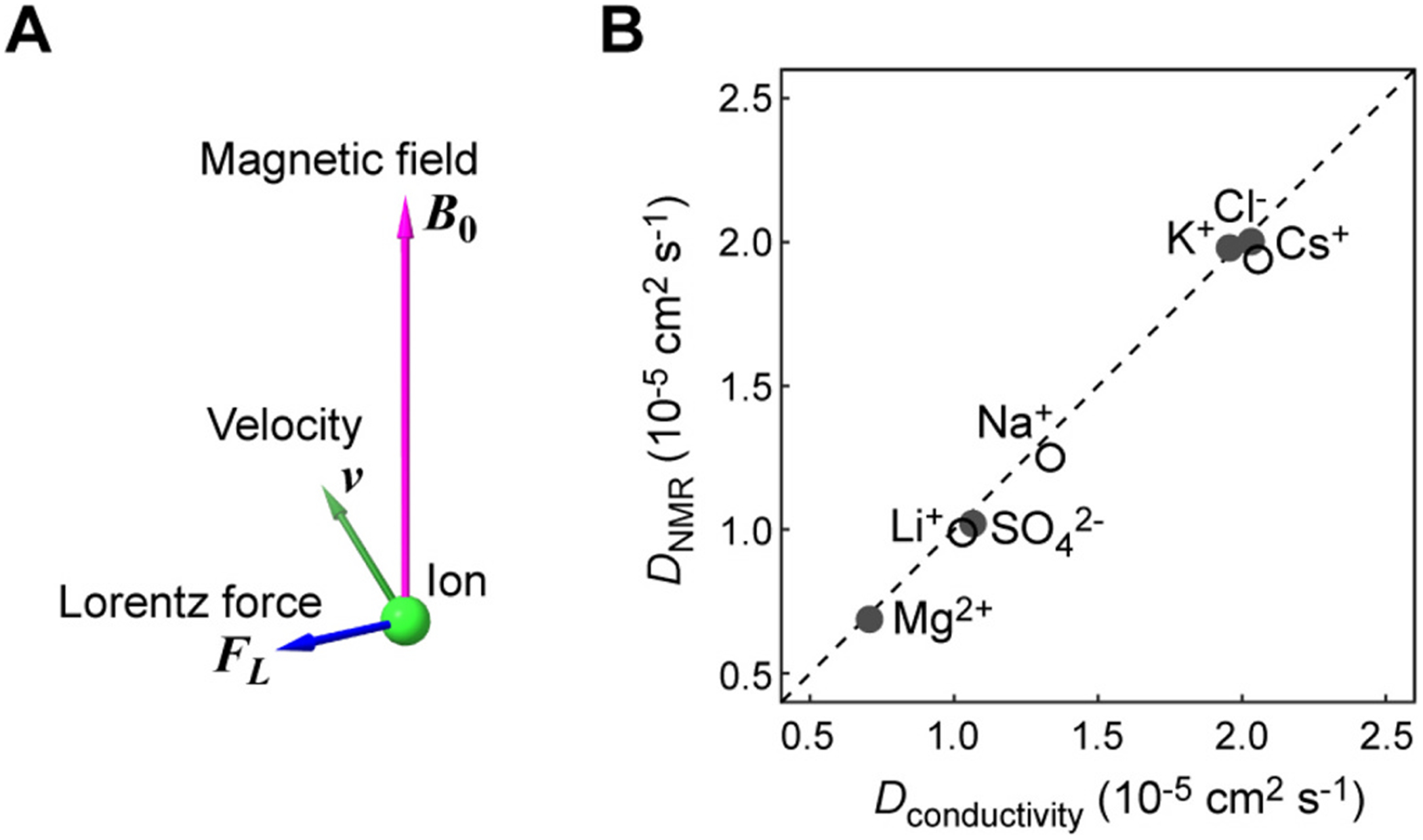
NMR-based measurements of ion diffusion along the *z* dimension are unaffected by the Lorentz force. (**A**) Pictorial representation of the Lorentz force acting on an ion. While the Lorentz force fluctuates due to random changes in the velocity through stochastic Brownian motion, this force (***F***_***L***_ = *q****v***×***B***_**0**_) is always perpendicular to the magnetic field. (**B**) Correlation between the ion diffusion coefficients measured by the NMR-based method and those measured by the conductivity-based method. The conductivity-based data are from the literature [48] and in the absence of any strong external magnetic field, whereas all of the NMR data were obtained at the magnetic field of 17.6 T. The NMR-based diffusion data are from the current study (closed circles) and those from the previous studies (open circles) [11,13]. These data are for solutions at 25 °C in the absence of any macromolecules.

Given that our studies on ions employ a high magnetic field, one might wonder if the Lorentz force has any impact on diffusion measurements by NMR. The Lorentz force ***F_L_*** acts on any moving charged particle and is given by *q****ν***×***B*_0_**, where *q* is the electric charge; ***ν*** is the vector of velocity; and ***B_0_*** are the magnetic field vector. As this expression indicates, the Lorentz force is perpendicular to both ***ν*** and ***B_0_*** (Fig. 3A) and is more pronounced with a greater electric charge at a higher magnetic field. Since ions undergo Brownian motions in solution, the velocity vector ***ν*** changes randomly, and consequently, the Lorentz force also fluctuates.

To consider if the Lorentz force can possibly impact the diffusional properties of ions in our high-field (17.6 T) experiments, estimating the magnitudes of the Brownian and Lorentz forces is helpful. Based on statistical mechanics, we can qualitatively compare the magnitudes of the Brownian force ***F_B_*** and the Lorentz force ***F_L_***, although both fluctuate. According to the fluctuation dissipation theorem, the mean squared Brownian force ${\left.\langle |F_{B}\right|}^{2}\rangle$ is given by $2\zeta k_{B}T$, where *k_B_* is the Boltzmann constant; *T* is absolute temperature; and ζ is the friction [44]. For a spherical particle, Stokes’ law and the Stokes-Einstein relation give ζ=6πηa and $D=k_{B}T/(6\pi \eta a)$ where *a* is the hydrodynamic radius and *η* is the viscosity. These are approximate when the size of the particle is comparable to the size of solvent molecule [45]. With this approximation, the average magnitude of the Brownian force |***F_B_***|*_ave_* can be estimated with ${\left|F_{B}\right|}_{\text{ave}}$ can be estimated with ${{\left.\langle |F_{B}\right|}^{2}\rangle }^{1/2}={\left(12\pi \eta ak_{B}T\right)}^{1/2}=(2/D{)}^{1/2}k_{B}T$. The Lorentz force on an ion is given by *q****ν***×***B*_0_**, where ***B*_0_** is the magnetic field; ***ν*** is the velocity; and *q* is the electric charge valence. While the velocity ***ν*** randomly fluctuates, the mean squared velocity ${\left.\langle |v\right|}^{2}\rangle$ based on the equipartition theorem for a three-dimensional space is given by 3*k_B_T*/*m* [44], where *m* is the mass. Using ${{\left.\langle |v\right|}^{2}\rangle }^{1/2}$ along with the electric charge and magnetic field, the average magnitude of the Lorentz force on the ion ${\left|F_{L}\right|}_{\mathrm{ave}}$ can be estimated to be $(2/\pi )qB_{0}{\left(3k_{B}T/m\right)}^{1/2}$, where the factor of 2/π is due to averaging for randomly oriented ***ν*** with respect to the magnetic field.

These estimates are useful for considering the relative magnitude of the Lorentz force with respect to the Brownian force. As a divalent ion with a relatively small mass, Mg^2+^ is subject to the largest average magnitude of the Lorentz force among the four ions investigated in our current study. For a Mg^2+^ ion under the current experimental conditions (*B*_0_ = 17.6 T and 25 °C), the estimated average forces are ${\left|F_{L}\right|}_{\mathrm{ave}}=0.06$ fN and ${\left|F_{B}\right|}_{\mathrm{ave}}=0.22fN$. Thus, the Lorentz force is significantly weaker but is not negligible compared to the Brownian force under our current experimental conditions.

Theoretical studies predicted that the Lorentz force affects the diffusion of charged particles on a plane perpendicular to the magnetic field but does not influence diffusion along the magnetic field [46,47]. This can be understood qualitatively, considering that the Lorentz force is perpendicular to the magnetic field. Since our diffusion NMR probe hardware is specifically designed to measure diffusion along the direction of the magnetic field (i.e., in the z dimension), the theoretical prediction suggests that our diffusion measurements of ions are unaffected by the Lorentz force. However, without experimental evidence, one cannot completely rule out the possibility that the Lorentz force somehow affects the apparent diffusion measurements, especially when the magnitude of the Lorentz force is large due to a high magnetic field and/or a larger electric charge (e.g., Mg^2+^ and ${\text{SO}}_{4}^{2-}$ ions) like our current case.

To examine whether the Lorentz force affects NMR-based diffusion measurements at 17.6 T, we compared our data with the conductivity-based ion diffusion coefficient data at 25 °C in the literature [48]. Unlike NMR-based measurements, conductivity-based diffusion measurements are possible only for simple salt solutions and are inapplicable to complex solutions involving several solutes. In this method, ion diffusion coefficients are determined from ion conductivities at infinite dilution, which are extrapolated from the concentration-dependent conductivity data for dilute (≤ 0.1 M) salt solutions [48]. Since the diffusion coefficients are only weakly dependent on salt concentration for dilute solutions [49], the conductivity-based ion diffusion coefficients are useful to compare with our NMR-based ion diffusion data. In the correlation plot shown in Fig. 3B, the K^+^, Mg^2+^, Cl^−^, and ${\text{SO}}_{4}^{2-}$ diffusion coefficients measured at 17.6 T and 25 °C by NMR in the current study are compared with those measured by conductivity-based approach. This figure also plots the corresponding NMR data for Na^+^, Li^+^, and Cs^+^ ions at 17.6 T and 25 °C from previous studies [11,13]. The NMR-based diffusion coefficients agree well with the conductivity-based diffusion coefficients within 8 % differences, supporting that the NMR-based diffusion measurements of ions along the *z* dimension are unaffected by the Lorentz force.

## 4. Discussion

Prior to our current work, there were some previous studies on ^25^Mg, ^35^Cl, and ^39^K diffusion NMR measurements [50–52]. However, these studies used either very high concentrations over 1 M (for ^39^K^+^ and ^25^Mg^2+^ ions) [51,52] or ions that exhibit relatively slow NMR relaxation (i.e., ^35^ClO^−^_4_ for which *T*_1_ = *T*_2_ = 220 ms) [50]. For example, although Graham et al. were able to determine ^39^K^+^ diffusion coefficients using PFG strengths up to 50 G/cm with a 11.7-T magnet [52], their approach requires a high concentration and is limited to conditions where K^+^ ions exhibit slow ^39^K NMR relaxation. They used the diffusion delay (Δ) of 135 ms and [K^+^] > 2 M. Their approach is inapplicable to K^+^ ions in biomolecular solutions at a biochemically meaningful concentration (< 0.2 M). In our current case, [K^+^] was 0.1 M, and K^+^ ions in 1 mM 12-bp DNA solution exhibit *T*_1_ and *T*_2_ relaxation times <26 ms. Since the delay Δ of 135 ms used by Graham et al. is more than 5 times the relaxation times, the ^39^K NMR signals from our DNA solution would decay completely in their stimulated-echo schemes. In this case, achieving sufficient dephasing with 50 G/cm through use of longer PFGs is not practically possible because PFGs with the maximum amplitude are typically limited to ~10 ms, which also causes a substantial decay due to the rapid ^39^K relaxation.

As demonstrated above, ions in biomolecular solutions exhibit far faster NMR relaxation due to interactions with biomolecules. The fast quadrupolar relaxation severely limits the practically available lengths of the stimulated-echo schemes for diffusion measurements. Since quadrupolar nuclei exhibit rapid relaxation for longitudinal magnetization as well, a long delay for longitudinal magnetization cannot be used to compensate for rapid relaxation of transverse magnetization. Thus, strong field gradients at a high magnetic field are essential for diffusion measurements of low-γ quadrupolar nuclei such as ^25^Mg, ^33^S, ^35^Cl, and ^39^K that exhibit rapid relaxation.

In previous studies, diffusion of counterions around nucleic acids and proteins was measured for Na^+^, Li^+^, Cs^+^, ${\text{NH}}_{4}^{+}$, and acetate (OAc^−^) ions using ^23^Na, ^7^Li, ^133^Cs, ^15^N, and ^13^C NMR spectroscopy [10–13]. These studies demonstrated that the diffusion coefficients of counterions bound to biomolecules are significantly larger than those of the biomolecules themselves. This finding suggests that counterions dynamically change their locations within the ion atmosphere [8,9]. When a protein binds to DNA, some counterions are released, contributing to the entropic term of the binding free energy [53,54]. The entropic effects of counterion release during protein-DNA association were studied for Na^+^, ${\text{NH}}_{4}^{+}$, and OAc^−^ ions using diffusion NMR spectroscopy [10–12]. With the probe hardware capable of generating strong field gradients, the same investigations are feasible for physiologically relevant K^+^, Mg^2+^, Cl^−^, and ${\text{SO}}_{4}^{2-}$ ions. This capability is significant, particularly for the divalent ions, as very little is known about their diffusional properties as counterions of biomolecules.

## 5. Conclusions

We have demonstrated that high-field diffusion probe hardware capable of generating strong field gradients enables NMR-based diffusion measurements of K^+^, Mg^2+^, Cl^−^, and ${\text{SO}}_{4}^{2-}$ ions in biomolecular solutions. The issue arising from small nuclear gyromagnetic ratios and rapid NMR relaxation of the quadrupolar ^39^K, ^25^Mg, ^35^Cl, and ^33^S nuclei were resolved through high-field experiments with strong field gradients (~1000 G/cm) that allow for sufficient dephasing via short stimulated-echo schemes. Our data also assure that the Lorentz force does not adversely impact the diffusion of ions along the *z* dimension at a high magnetic field. Thus, high-field NMR probe hardware capable of generating strong magnetic field gradients opens a new avenue to characterize the dynamic behavior of various ions around biomolecules. This method allows for investigations of electrostatic interactions of ions with biomolecules and is complementary to the paramagnetic NMR methods that allow for direct measurements of electrostatic potentials around biomolecules [55,56]. The new capability of measuring diffusion for K^+^, Mg^2+^, Cl^−^, and ${\text{SO}}_{4}^{2-}$ ions in biomolecular solutions will facilitate biophysical research on the role of ions in biomolecular processes.

## Figures and Tables

**Table 1:** Properties and natural abundance of the nuclei relevant to this study.^a)^

Nucleus	Spin quantum number	Gyromagnetic ratio *γ* (10^7^ rad s^−1^ T^−1^)	|*γ*X/*γ*H|	Natural abundance (%)
^1^H	1/2	26.75220	1.000	99.99
^25^Mg	5/2	−1.63890	0.061	10.13
^33^S	3/2	2.05568	0.077	0.76
^35^Cl	3/2	2.62420	0.098	75.53
^39^K	3/2	1.24993	0.047	93.10

a From Ref. [19].

## Data Availability

Data will be made available on request.
